# Characterization of Natural Bioactive Compounds from Greek Oregano Accessions Subjected to Advanced Extraction Techniques

**DOI:** 10.3390/plants13213087

**Published:** 2024-11-02

**Authors:** Christina Panagiotidou, Elisavet Bouloumpasi, Maria Irakli, Paschalina Chatzopoulou

**Affiliations:** Hellenic Agricultural Organization—Dimitra, Institute of Plant Breeding and Genetic Resources, P.O. Box 60458, 57001 Thessaloniki, Greece; cristinapanagiotidou@hotmail.com (C.P.); ebouloum@vo.duth.gr (E.B.)

**Keywords:** *Origanum vulgare*, microwave-assisted extraction, ultrasound-assisted extraction, accelerated solvent extraction, antioxidant, LC-MS, rosmarinic acid, phenolic compounds

## Abstract

Nowadays, eco-friendly extraction techniques are often used to develop natural plant extracts for commercial use. In the current investigation, Greek oregano (*Origanum vulgare*) phenolic extracts from different cultivated accessions were recovered employing ultrasonic-assisted extraction (UAE), microwave-assisted extraction (MAE), and accelerated solvent extraction (ASE). The phytochemical profile of the oregano extracts, as determined by spectrophotometric and chromatographic techniques, as well as antioxidant activity (ABTS, DPPH, and FRAP assays), was used to compare the three extraction approaches. The results showed that oregano phenolic extracts obtained by MAE held the highest total phenolic content, total flavonoid content, and also a higher content of the main phenolic compounds identified, rosmarinic acid, salvianolic acid B and carvacrol, as determined by LC-MS analysis, followed by those extracted by UAE and ASE. All of the tested extracts exhibited relatively high antioxidant activities, especially the MAE extracts. Oregano extracts produced by various extraction techniques were subjected to a multivariate data analysis to highlight differences in phytochemical profiles, and their correlation to antioxidant activity. According to our findings, it was evident that MAE offers more efficient and effective extraction of bioactive compounds in terms of obtaining phytochemical-rich oregano extracts, with applications in the food and pharmaceutical industries.

## 1. Introduction

Oregano *(Origanum vulgare* L.), belonging to the family Lamiaceae, is widely distributed through the Mediterranean area and is also found in many parts of Europe, Asia, and North Africa. Due to its specific flavor, owed to the contained essential oil, oregano has been used since antiquity as a spice, being typical of Mediterranean cuisine, flavoring different foods and dishes, and possessing a dominant role among culinary herbs worldwide. Additionally, oregano represents a highly important natural resource widely used as traditional medicine for the treatment of indigestion, asthma, diarrhea, and rheumatoid arthritis, while numerous studies have reported its antimicrobial, antifungal, antioxidant, carminative, antispasmodic, and anti-inflammatory properties [1,2]. The *O. vulgare* species is regarded to be extremely variable, and according to the latest accepted taxonomic revision [3], six subspecies have been recognized, based on morphological traits [4]. Besides that, great variability in the content and composition of oregano essential oils produced from the different subspecies has been reported; those obtained from southern-grown taxa are essential oil-rich, though those from North and Central Europe are poor [5]. In Greece, three subspecies occur, and among them, the ssp. *hirtum* (Link) Ietswaart, known as “Greek oregano”, is the most commercial due to its high content of essential oil and its main constituent carvacrol (55–94%) [6]. However, different ecotypes and populations have been reported to produce various amounts of essential oils and ratios of the phenolic terpenes carvacrol and thymol, thus presenting different chemotypes [6,7,8].

Due to the increasing interest of consumers in natural antioxidants, oregano has been the subject of numerous studies due to its antibacterial and antioxidant activities [9,10,11,12], attributed to the high content of phenolics in essential oils and extracts. Phenolic compounds are important, contributing to lipid peroxidation stabilization and inhibiting various oxidizing enzymes [10], thus improving the quality and extending the shelf life of food products. Previous studies have shown that Greek oregano extracts are effective against free radicals and lipid oxidation [13,14,15]. On the other hand, natural antioxidants may reduce oxidative stress due to their radical-scavenging activity, exhibiting health benefits for several chronic pathologic conditions associated with free radicals.

Besides essential oils, oregano contains considerable amounts of hydrophilic phytochemicals, the most important being flavonoids and phenolic acids. Gutiérrez-Grijalva et al. [16] have reported that the most important phenolic acids and flavonoids in oregano species are rosmarinic acid, apigenin, luteolin, quercetin, scutellarein, and their derivatives. The constitution of Greek oregano extracts has been investigated by several authors; carvacrol and rosmarinic acid were the major compounds, and taxifolin, eriodictyol, apigenin, naringenin, luteolin, and kaempferol were also detected [2,15,17,18].

Extraction is a crucial step in isolating bioactive compounds from plants. This process transforms the raw plant material into a sample ready for further analytical procedures. Various traditional extraction methods such as decoction, maceration, infusion, digestion, percolation, and Soxhlet have been used to prepare natural extracts from medicinal plants, which are known to be time-, solvent-, and energy-intensive [19]. In contrast, various non-conventional extraction methods, such as microwave-assisted extraction (MAE), ultrasound-assisted extraction (UAE), accelerated solvent extraction (ASE), and many others such as supercritical fluid extraction (SFE), pulsed electric fields-assisted extraction (PEF), etc [20,21,22,23,24], have been developed to increase the yield of bioactive compounds from aromatic plants and minimize the disadvantages of the conventional techniques. This has led to a growing demand for sustainable choices for high-quality extraction with environmentally friendly approaches. Consequently, they fall under the category of ‘Green Extraction’ techniques, as they offer sustainable and efficient alternatives to conventional methods, allowing for increased extract yield, shorter extraction times, and low energy cost [25]. The extraction of phenolic compounds from *Origanum vulgare* ssp. *hirtum* by different environmentally friendly techniques [26,27,28] has been reported. Nonetheless, comparative studies between UAE, MAE, and ASE on extracting polyphenols from cultivated Greek *Origanum vulgare* accessions are limited.

Taking into consideration the reputation of oregano as a natural antioxidant and its potential exploitation as functional foods, the great genetic and chemical variability among different populations, and the urgent need to decrease the consumption of energy and produce eco-friendly natural products, the present study aims to evaluate the effect of three advanced extraction methods (MAE, UAE, and ASE) on the phytochemical profile and antioxidant activity of four cultivated Greek oregano accessions and two commercial cultivations representing different chemotypes.

## 2. Results and Discussion

### 2.1. Optimization Study Through Single-Factor Experiments

**Figure 1:**
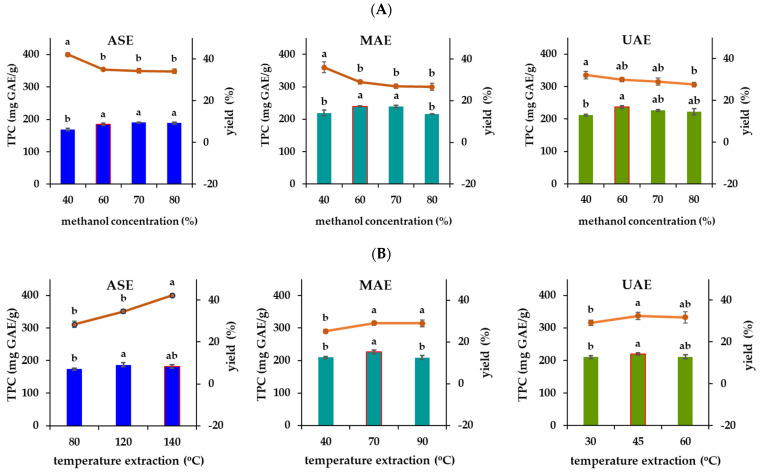
Effect of methanol concentration (%) (**A**) and extraction temperature (**B**) on yield and total phenolic content (TPC) of the oregano extracts. Different letters (a, b) among columns with the same color for each extraction method (ASE, MAE, UAE) indicate significant differences (*p* < 0.05) amongst the means, as determined by Tukey’s multiple comparison test; the data are means of three independent replicates.

In order to establish the optimal extraction parameters for each extraction technique used, the effect of two chosen extraction variables (methanol concentration and temperature extraction) on the extraction yield and total phenolic content (TPC) of oregano extracts was assessed by single-factor experiments. Methanol was selected as the extraction solvent, considering the polarity of the major compounds [29], polarity index, and dielectric constant of the solvent, as well as the previously published literature related to the phenolic compound extraction from oregano [30]. Firstly, the efficiency of different methanol concentrations (40, 60, 70, and 80% *v*/*v*) was studied by using ASE, MAE, and UAE to determine the extraction yield and TPC of oregano extracts (Figure 1A). The extraction yield and TPC showed a significant variation depending on the methanol concentration used as well as the extraction technique applied. The results indicated that the extraction yield gradually decreased with an increase in methanol percentage in the extraction solvent from 40 to 80% (Figure 1A). However, TPC displayed a significant increase with an increase in methanol concentration from 40 to 60%, while no significant difference was present with a greater increase in methanol concentration up to 80%, except for with MAE (Figure 1A). As the TPC is an index of the antioxidant capacity of oregano extracts, we decided to focus more on TPC than extraction yield of obtained oregano extracts; therefore, 60% *v*/*v* was selected as the optimum methanol concentration for further experiments under all extraction methods (ASE, MAE, UAE).

The effect of temperature on the yield and TPC of oregano extracts appeared to be dependent on the extraction method used (Figure 1B). Specifically, increasing the ASE temperature from 80 °C to 140 °C was noted to result in an increase in yield and TPC, so the upper level was selected as the optimum extraction temperature under ASE. The effect of MAE and UAE temperature on the yield and TPC of oregano extracts presented a similar trend. Concerning the effect of temperature in MAE, temperature increases up to 70 °C favored the extraction of the phenolic compounds present in the oregano, while higher increases in temperatures up to 90 °C led to a significant decrease in TPC. Similarly, increasing the UAE temperature from 30 to 45 °C had a significant favorable impact on yield and TPC, while further raising the temperature resulted in a negligible drop in these parameters. These findings are in accordance with those reported by a previous study [30], which reported that higher temperatures favor extraction by increasing the solubility and diffusion coefficient of phenolic compounds from oregano. According to our findings, 90 and 45 °C were selected as optimum extraction temperatures for MAE and UAE, respectively.

### 2.2. Total Phenolic Content (TPC) and Total Flavonoid Content (TFC) of Different Oregano Extracts

In the present work, three advanced extraction methods (ASE, MAE, and UAE) were applied for the isolation of phenolic compounds from six Greek oregano accessions, representing different essential oil chemotypes (with various ratios of carvacrol/thymol). The analysis of variance (ANOVA) showed that all sources of variation were highly significant in all antioxidant-related traits (Table 1).

The extraction method employed and oregano accession used showed highly significant effects (*p* < 0.001) on all investigated traits, while interactions between both parameters (*p* < 0.05) showed highly significant effects (*p* < 0.001) on FRAP values, moderately significant effects (*p* = 0.005) on DPPH values, and low (*p* < 0.05) significant effects in TPC and TFC values.

**Figure 2:**
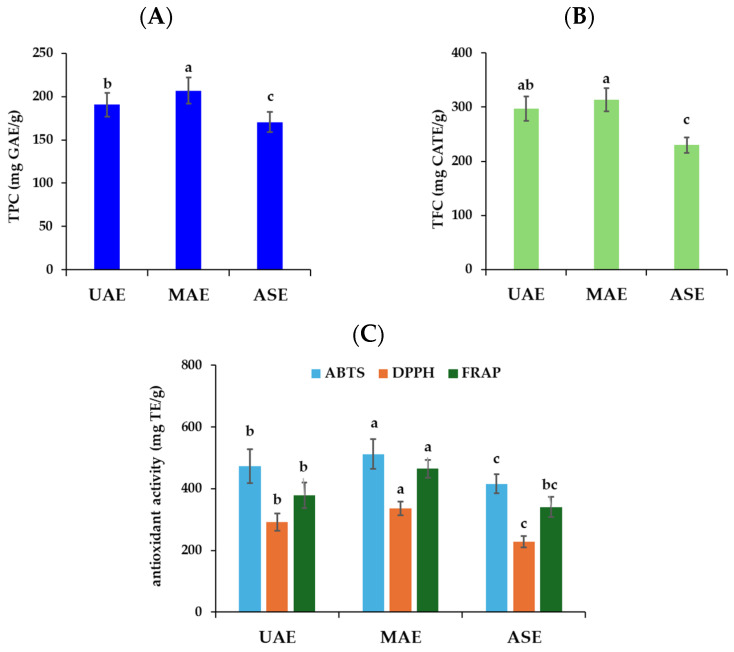
Effect of UAE, MAE, and ASE on (**A**) total phenolic content (TPC), (**B**) total flavonoid content (TFC), and (**C**) antioxidant activity of the oregano phenolic extracts (mean value of six oregano accessions) as evaluated by 2,2′-azinobis-(3-ethylbenzothiazoline-6-sulfonic acid radical-scavenging activity (ABTS), 2,2-diphenyl-1-picrylhydrazyl radical-scavenging activity (DPPH) and ferric-reducing antioxidant power (FRAP), corresponding to mean values of six oregano accessions. Different letters among columns with the same color indicate significant differences (*p* < 0.05) among the extraction methods (UAE, MAE, ASE), as determined by Tukey’s multiple comparison test.

Oregano extracts obtained by MAE exhibited the highest TPC, ranging from 195.0 to 232.1 mg GAE/g among the different studied accessions, with a mean value of 206.9 ± 15.1 mg GAE/g (Figure 2A). UAE was the second most efficient method, achieving extracts with TPC values varying from 178.0 to 214 mg GAE/g, with a mean value of 190.6 ± 13.6 mg GAE, whereas ASE appeared to be the least efficient extraction technique with TPC ranging from 156.0 to 182.9 mg GAE/g and a mean value of 170.6 ± 11.5 mg GAE/g (Figure 2A). Similarly, Calderon Oliver et al. [19] reported that MAE was the most efficient method to obtain oregano extracts, followed by UAE, Soxhlet, and agitation. Likewise, Nile et al. [31] found that ethanolic extracts of *O. vulgare* acquired by MAE had the greatest TPC (65.40 mg GAE/g), followed by extracts obtained by UAE (56.22 mg GAE/g), Soxhlet (50.88 mg GAE/g), and maceration (46.89 mg GAE/g). On the contrary, Zengin et al. [32] showed that the *O. vulgare* subsp. *viridulum* extract obtained by ASE had the highest phenolic content (186.63 mg GAE/g), in comparison to conventional (Soxhlet, maceration) and novel extraction techniques (MAE, UAE), followed by MAE (170.90 mg GAE/g). Meanwhile, Giannenas et al. [33] reported lower TPC (52.1 mg GAE/g dw) for Greek oregano extract acquired by UAE, probably due to the specific chemotype used.

Although slight differences occurred between the different oregano accessions (Table 1), there were significant differences in extraction techniques regarding the TPC, following the order MAE > UAE > ASE. Among the oregano accessions, A4 showed the highest TPC in MAE, UAE, and ASE extracts (232.1, 214.0, and 182.9 mg GAE/g, respectively) possibly due to its higher content of carvacrol compared to other studied rich carvacrol accessions (A3, C2, C1).

The results regarding the TFC are also represented in Table 1 and Figure 2B. It has been noticed that the highest TFC was recorded for MAE extracts (314.1 ± 21.3 mg CATE/g) and the lowest in ASE extracts (229.8 ± 13.8 mg CATE/g); however, no significant difference occurred between the MAE and UAE extracts. Comparing the similar extraction techniques applied for *O. vulgare* subsp. *Viridulum.,* Zengin et al. [32] showed that TFC was higher in extracts obtained by ultrasonication, followed by MAE and ASE. Concerning the effect of chemotype, the C1 oregano accession demonstrated the highest TFC (331.3 mg CATE/g) in MAE extracts, without being significantly different from the A1, A3, and A4 accessions, whereas the lowest value was detected in the A2 sample (272.8 mg CATE/g). High TFC values were also detected in UAE extracts. Similarly, A3 and C1 samples were the most abundant in TFC, followed by C2, A1, and A4, whereas the lowest was detected in A2. The same trend was observed in oregano extracts obtained by the ASE method. The A3 accession was the richest in TFC, albeit with reduced content as compared to MAE and UAE, followed by C2, C1, and A1, while the lowest one was observed in A2, similarly to previous methods.

### 2.3. Antioxidant Activity of Different Oregano Extracts

The assessment of antioxidant potential is crucial for using an extract in different food systems and illustrates how these bioactive substances may protect cells from oxidative damage. When oxidants and antioxidants are not in balance, oxidative stress ensues and is linked to a number of significant illnesses and disorders. Plant-derived bioactive substances, such as flavonoids and phenolics, possess many functions; therefore, it is crucial to evaluate their biological activities using a variety of assays. In this study, the antioxidant activity of the oregano extracts obtained by MAE, UAE, and ASE was evaluated by three assays: ABTS radical-scavenging activity, DPPH radical-scavenging activity, and ferric-reducing antioxidant power (FRAP). As shown from the results presented in Table 1 and Figure 2C, MAE proved to be the most effective method to acquire oregano extracts with the highest antioxidant potential, followed by UAE and ASE. Similarly, other researchers [19,34,35] have shown that the MAE method is superior for extracting phenolic compounds with antioxidant potential, including rosmarinic acid, from plants of the Lamiaceae family, like oregano, thyme, rosemary, and peppermint. However, Zengin et al. [32] showed that *O. vulgare* extract obtained by ASE had the highest potential in the FRAP (1433.94 mg TE/g) assay, as well as the highest capacity towards DPPH (359.45 mg TE/g) and ABTS (679.63 mg TE/g) radicals, followed by those obtained by the MAE method, possibly due to the different constitution of polyphenols in this species compared to our sample (*O. vulgare* subsp. *hirtum*).

Oreopoulou et al. [36] reported that 60% ethanol is the most efficient extraction means for phenolic recovery from post-distillation Greek oregano essential oil residues, achieving the strongest antiradical capacity. In our case, the MAE method, which exhibited the highest antioxidant activity, was applied using 60% methanol, similar to the UAE and ASE methods. Other studies confirm that 50–70% ethanol in water appears to be the greatest choice for extracts that are high in bioactive substances and have excellent antioxidant activity [37,38].

Concerning the effect of different essential oil chemotypes, the antioxidant activity decreased in the extracts obtained by the three methods, in the order A4 > A3 > C2 > C1 > A2 > A1 in the ABTS method, closely correlated to the content of carvacrol in oregano EO. Similarly, extracts of A3, A4, C1, and C2, obtained by the three techniques, exhibited the highest antioxidant potential measured by the FRAP method; however, in UAE and ASE extracts, the antioxidant activity of A1—being a different chemotype—was not significantly different from C1 and C2. The A3 accession had the highest antioxidant activity in extracts obtained by MAE and UAE, according to the DPPH assay; however, A4 was more powerful in extracts obtained by ASE, with A3 and A2 coming in second. It should be noted that the antioxidant properties of medicinal plants are mostly attributed to phenolic compounds. Certain plant species, however, contain additional antioxidant compounds other than phenolics, and plant extracts frequently comprise intricate blends of various active compounds, so it is important to acknowledge the role played by compounds other than phenolics [31].

### 2.4. Phenolic Profile of Different Oregano Extracts

**Figure 3:**
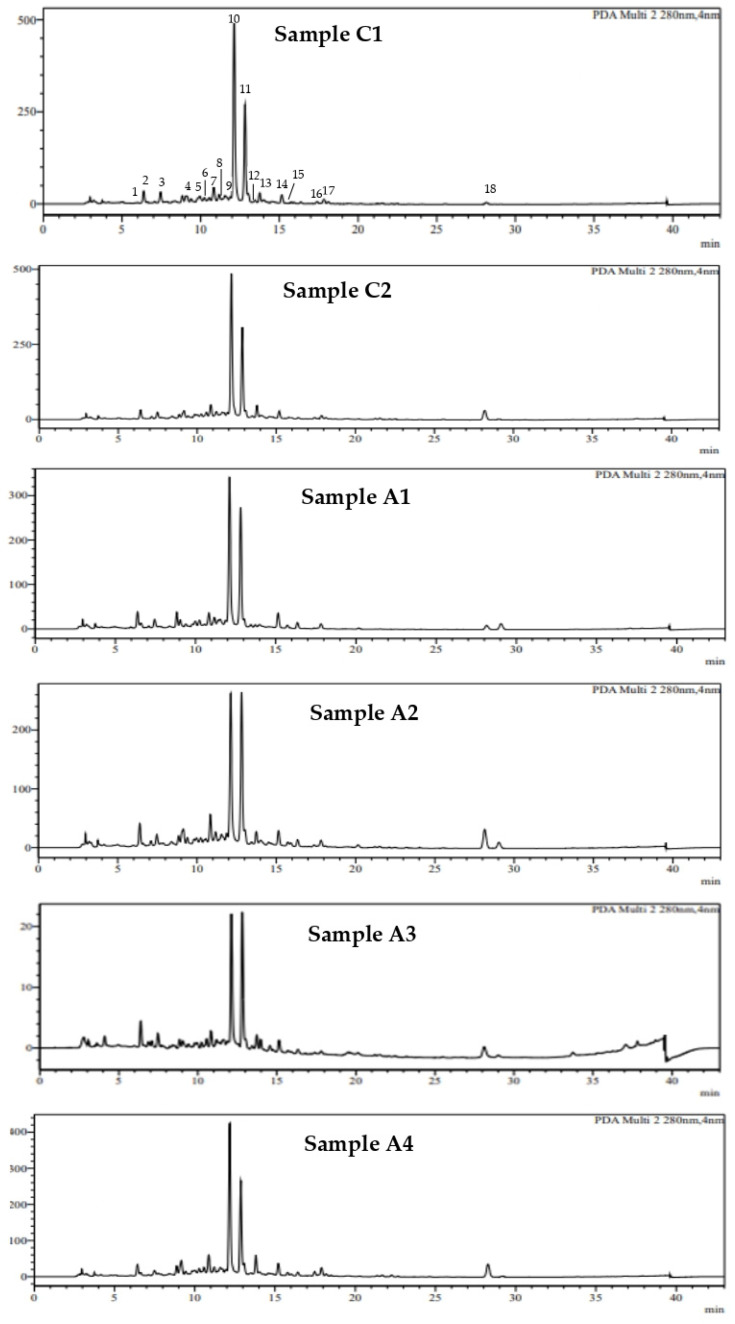
HPLC chromatogram at 280 nm of the oregano extracts obtained from the two commercial cultivations (C1, C2) and the four accessions (A1, A2, A3, A4) by using MAE. Peaks on the chromatogram correspond to the following: 1, crypto-chlorogenic acid; 2, vicenin-2; 3, caffeic acid; 4, salvianolic acid isomer I; 5, salvianolic acid isomer II; 6, verbascoside; 7, taxifolin; 8, salvianolic acid isomer III; 9, apigenin-7-O-glucoside; 10 rosmarinic acid; 11, salvianolic acid B; 12, aromadendrin; 13, salicylic acid (internal standard); 14, eriodictyol; 15, luteolin; 16, apigenin; 17, naringenin; 18, carvacrol.

The MAE, UAE, and ASE extracts of the different oregano accessions were analyzed by LC–DAD-MS. Representative chromatograms of the studied oregano extracts obtained under MAE are illustrated in Figure 3. The main compounds identified in all oregano extracts were rosmarinic acid (peak 10), salvianolic acid B (peak 11), carvacrol (peak 18), and vicenin-2 (peak 2), while salvianolic acid isomers (I, II, and III); flavonoids, namely eriodictyol, taxifolin, naringenin, aromadendrin, apigenin, luteolin, and apigenin-7-O-glucoside; and phenolic acids, namely caffeic acid and crypto-chlorogenic acid, were detected in lesser amounts. Similarly, Tsimogiannis et al. [15] identified taxifolin, aromadendrin, eriodictyol, apigenin, carvacrol, rosmarinic acid, and apigenin glycoside in diethyl ether and ethanol extracts of *O. vulgare* subsp. *heracleoticum*, syn subsp. *hirtum*. Oreopoulou et al. [36] studied the kinetics of conventional hydroalcoholic extraction of post-distillation Greek oregano essential oil residues, and detected rosmarinic acid, lithospermic acid (salvianolic acid B), flavonoid glycosides, and carvacrol as the major phenolics. Similarly, Bouloumpasi et al. [39] detected numerous phenolics in the extracts of Greek oregano distillation solid residues, mainly rosmarinic acid and salvianolic acid isomers. According to Exarhou et al. [40], the predominant component of ethanol extracts of *Oregano vulgare* L. ssp. *hirtum* was rosmarinic acid.

Frías-Zepeda [41] reported that ethanolic extracts of Mexican oregano (*Lippia graveolens*) leaves, after the essential oil removal, contained caffeic acid, naringenin, taxifolin, eriodictyol, acacetin, luteolin, quercetins-3-O-glycoside, apigenin, floridzin, and quercetin. Jafari Khors et al. [12] found that the main compounds of methanolic extracts of three *Origanum vulgare* subspecies, cultivated in Iran, were rosmarinic acid, which was the major one, ranging from 659.6 to 1646.9 mg/100 g dw, followed by luteolin (46.5–345.4 mg/100 g dw), quercetin, and naringenin. Yan et al. [1] found that *O. vulgare* subsp. *hirtum* was the richest in rosmarinic acid, compared to subsp. *vulgare*, subsp. *viride*, subsp. *glacile,* and subsp. *virens*. Similarly, Baranauskaite et al. [2] revealed that the ethanolic extract of *O. vulgare* subsp. *hirtum* contained higher amounts of rosmarinic acid, compared to *O. onites*, though carvacrol was most abundant in the latter. Different oregano species may have quite distinct flavonoid and phenolic compound compositions. Furthermore, it has been shown that the primary determinants of the flavonoid and phenolic acid profile of chemotypes within a single species are both geographical and environmental factors [41].

**Figure 4:**
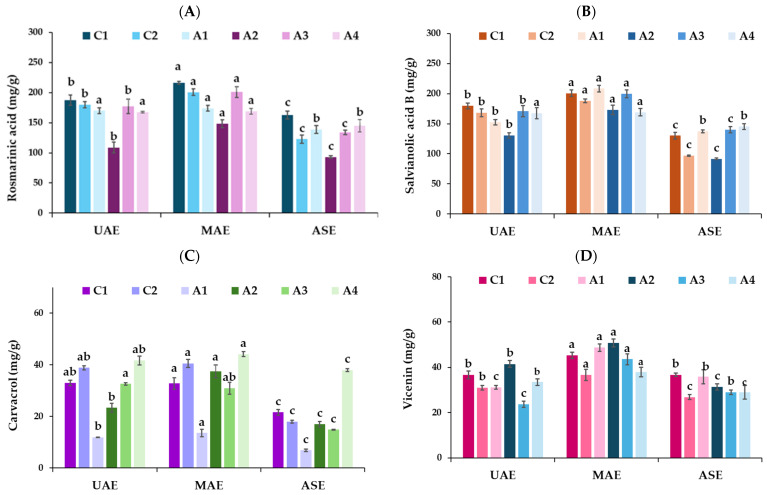
Effect of different extraction processes on the main compounds of the studied oregano extracts: (**A**) rosmarinic acid, (**B**) salvianolic acid B, (**C**) carvacrol, and (**D**) vicenin. Different letters among columns with the same color indicate significant differences (*p* < 0.05) among the extraction methods (UAE, MAE, ASE).

Rosmarinic acid was recovered from the studied accessions roughly equally by the MAE and UAE procedures (mean values not statistically different) and in significantly higher amounts compared to the ASE technique, as illustrated in Figure 4A. Rosmarinic acid is an abundant phenolic compound in many genera of the Lamiaceae family, known for its bioactivity and anti-inflammatory, antioxidant, hepatoprotective, antitumor, and antiviral properties [42]. In general, the A2 accession contained the lowest amount of rosmarinic acid in all extracts obtained by the MAE, UAE, and ASE techniques. According to Exarchou et al. [40], although the amounts of rosmarinic acid and caffeic acid in oregano species are clearly considerable, there is a significant variation in the relative quantities of these two phenolic acids among the subspecies.

Salvianolic acid B, one of the main phenolic compounds in oregano, known for its biological activity [43], was extracted in higher amounts through MAE, without being significantly different among the studied accessions (Figure 4B). UAE was the second most efficient method for salvianolic acid B recovery, whereas in ASE extracts, it was significantly lower than in MAE and UAE extracts. 

He et al. [44] reported that UAE yielded higher salvianolic acid B than the conventional refluxing method, and the procedure was executed under a shorter time and lower temperature. The same researchers reviewed the numerous pharmacological properties of salvianolic acid B, including anti-inflammatory, antioxidant, antitumor, and protective effects on various diseases. Carvacrol is the characteristic phenolic compound of oregano plants and the major constituent of its essential oil, with its amount depending on several factors: genotype, growing conditions, ontogenetic stage, etc. In this study, oregano accessions representing different essential oil chemotypes and different ratios of carvacrol/thymol, cultivated under the same conditions (A1, A2, A3, and A4), as well as commercial cultivations (C1, C2), were rich in carvacrol. The extracts obtained by MAE and UAE contained almost equal amounts of carvacrol that were significantly higher than those obtained by the ASE method (Figure 4C). More specifically, the A4 accession in MAE, UAE, and ASE extracts was the most abundant in carvacrol, in line with its high percentage in respective essential oils, whereas the A1 extract was the accession with the lowest carvacrol content, which was in accordance with the corresponding essential oil. In our findings, the ASE method revealed extracts with low carvacrol content in comparison to MAE and UAE extracts. As for the flavonoid compound vicenin, it was also extracted in higher amounts by the MAE method, whereas the UAE and ASE procedures did not show significant differences in its mean values (Figure 4D). Zengin et al. [32] determined that the most effective approach for extracting phenolic acids and their derivatives from *O. vulgare* was MAE, followed by Soxhlet and ASE. Instead, in this particular case, the maceration and UAE approaches have been shown to be more suitable techniques for extracting flavonoids and their derivatives.

The two-way analysis of variance (ANOVA) showed significant effects related to the extraction method employed (*p* < 0.001), the oregano accession (*p* < 0.001), and the interactions between both parameters (*p* < 0.001) on the contents of major phenolic compounds (Table 2).

Minor phenolic compounds quantified in all oregano samples were salvianolic acid I (0.87–25.72 mg/g), salvianolic acid II (0.74–15.78 mg/g), salvianolic acid III (5.58–14.70 mg/g), eriodictyol (3.17–8.67 mg/g), taxifolin (1.18–4.32 mg/g), naringenin (0.65–3.41 mg/g), aromadendrin (0.26–1.30 mg/g), apigenin (0.15–0.67 mg/g), luteolin (0.19–0.74 mg/g), caffeic acid (0.43–2.53 mg/g), crypto-chlorogenic acid (0.09–1.72 mg/g), and apigenin-7-O-glucoside (0.09–0.61 mg/g) (Table 3), while trace amounts were identified for kaempferol. The highest value for all phenolic compounds quantified appeared in MAE extracts, with the exception of caffeic acid, in which ASE extracts yielded higher amounts than the MAE and UAE ones. The two-way analysis of variance (ANOVA) showed significant effects related to the extraction method employed, the oregano accession (*p* < 0.001), and the interactions between both parameters on the contents of minor phenolic compounds (Table 2).

### 2.5. Principal Component Analysis and Heatmap Analysis

**Figure 5:**
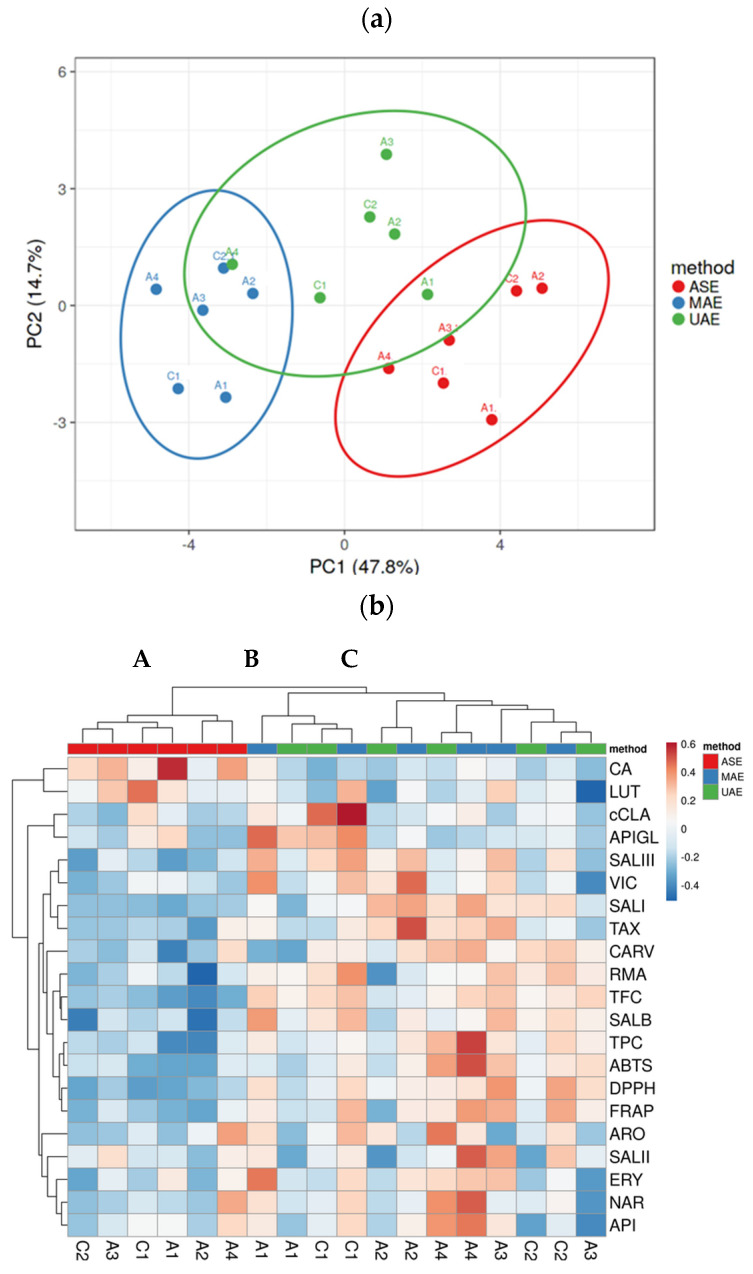
PCA plot of the first two components of MAE, UAE, and ASE extracts of four oregano accessions and two commercial cultivations (**a**) and heatmap visualization showing fold changes of the same traits in different extracts (**b**). A, B, C correspond to groups. Columns are clustered using Euclidean distance and Ward linkage (18 rows, 21 columns).

Multivariate analysis was performed with the content of 16 phenolic compounds determined by LC/MS, as well as with TPC, TFC, and antioxidant capacities (ABTS, DPPH, and FRAP values), as variables to compare MAE, UAE, and ASE extracts in four different oregano accessions and two commercial cultivations. In the principal component analysis (PCA), the score plot of the first two principal components accounting for 62.5% of the total variance (Figure 5a) showed a high variation within the green extracts and oregano samples and thus were not completely distinguishable in phenolic compositions and antioxidant activity between MAE, UAE, and ASE extracts.

The overlapping of clusters reflects similarities in composition. According to the score values of PCA, the studied oregano extracts obtained by ASE, MAE, and UAE were classified into three different groups. In particular, all ASE extracts could be well distinguished from MAE and UAE extracts, with the exception of the UAE extract of the A1 accession, which shows an overlap. In addition, the bioactive profiles of the MAE and UAE extracts showed non-noticeable differences among the oregano accessions and commercial cultivations. However, MAE extracts of A1 and A4 oregano accessions and the C1 commercial cultivation are well distinguished from the corresponding UAE extracts. 

As displayed in the heatmap (Figure 5b), the various oregano extracts can be divided into three groups: group A includes only ASE extracts, while group B combines both MAE and UAE extracts of A1 and C1 oregano accessions, whereas group C includes MAE and UAE extracts of other oregano accessions tested. Group C can be divided into three subgroups (a), (b), and (c). Subgroup (a) includes both MAE and UAE extracts of the A2 oregano accession, with a significant content of vicenin-2, taxifolin, and salvianolic acid isomers I and III, but a lower TFC, rosmarinic acid content, and antioxidant activity (blue). In subgroup (b), the UAE and MAE extracts of A4 have been grouped and characterized by a predominance of orange color, which indicates a high positive correlation between phenolic compounds (naringenin, apigenin, carvacrol, eriodictyol, aromadendrin, and salvianolic acid isomers I and II) and TPC as well as antioxidant activities based on ABTS, DPPH, and FRAP values. In addition, in subgroup (c), the A3 and C2 extracts are characterized by higher levels of rosmarinic acid, salvianolic acid B, and TFC (orange color).

Finally, results from the heatmap analysis were in accordance with the PCA. Significant variabilities among studied extracts can be explained by the extraction method used, which reflects the difference in their phenolic composition and the antioxidant activities as well. The heatmap allowed us to select the superior extract as the major antioxidant oregano accession. Correlation analysis showed some relations among phenolic compounds and antioxidant attributes (Table S1). There was a strong relationship between antioxidant values with TPC and TFC (0.761–0.885). Furthermore, correlation analysis revealed that rosmarinic acid and carvacrol contents were moderately correlated with ABTS and DPPH antioxidant activity (r = 0.517–0647, r = 0.534–0.797, r = 0.504–0.656, respectively), whereas high correlations were demonstrated between salvianolic acid B content and DPPH and FRAP antioxidant activity (r = 0.774, r = 0.797, respectively). Moreover, there were moderate correlations between antioxidant activity and eriodictyol, taxifolin, naringenin, aromadendrin, and apigenin (r = 0.387–0.647). However, there was not a significant correlation among luteolin, c-chlorogenic acid, apigenin-7-O-glucoside, and antioxidant activity, while there was a negative and significant correlation (*p* < 0.001) between caffeic acid content and antioxidant capacity.

## 3. Materials and Methods

### 3.1. Plant Materials

Aerial parts of Greek oregano (*O. vulgare* ssp. *hirtum*) were collected during full flowering in June 2021 from (i) cultivated accessions (A1, A2, A3, A4) in the experimental field of Hellenic Agricultural Organization—Dimitra—Institute of Plant Breeding and Genetic resources (Thermi, Thessaloniki) originating from different districts of Greece, representing different essential oil chemotypes, and (ii) from commercial cultivations in regions of Central Macedonia (C1, C2). Oregano accessions represented different essential oil chemotypes, containing diverse ratios of carvacrol/thymol, the main compounds in their essential oils (unpublished data), as follows: A1, 5.0/66.7%; A2, 53.9/22.0%; A3, 76.0/0.6%; A4, 82.3/0.2%; C1, 72.5/2%; C2, 76.5/1.90%. After drying at room temperature, flowers and leaves were separated from stalks and stored under controlled environmental conditions (25 °C) until extraction was carried out. The plant material was ground in a laboratory mill (Retsch, Model ZM 1000, Haan, Germany) to pass through a 0.5 mm sieve, and then it was stored at 4 °C until further analysis.

### 3.2. Chemicals

The analytical reagents 2,2-diphenyl-1-picryhydrazyl (DPPH), 2,2-azinobis-(3-ethylbenzthiazoline-6-sulphonic acid) (ABTS), and 2,4,6-tripyridyl-s-triazine (TPTZ) were purchased from Sigma-Aldrich (Steinheim, Germany). Analytical standards of phenolic compounds were purchased from Extrasynthese (Genay Cedex, France). All the solvents used for the extraction of phenolic compounds as well as the chromatographic analysis were of HPLC or LC-MS grade.

### 3.3. UAE

The oregano powdered material was weighed (0.1 g) into a double-walled extraction tube. The material was then subjected to extraction with 10 mL of aqueous methanol in varying concentrations (40, 60, 70, and 80% *w*/*w*), employing an ultrasonic bath (frequency 37 kHz, model FB 15051, Thermo Fisher Scientific Inc., Loughborough, UK) for a constant time of 15 min, at varying extraction temperatures (30, 45, and 60 °C). Following extraction, the samples were centrifuged at 10,000× *g* for 1 min at 4 °C. The supernatants were subjected to an evaporation process at 40 °C using a rotary vacuum distillation apparatus (Heidolph Instruments GmbH & Co. KG, Schwabach, Germany). The remaining aqueous extract underwent lyophilization (Christ, Martin Christ Gefriertrocknungsanlagen GmbH, Osterode am Harz, Germany) for 48 h and then the extracts were stored at −25 °C.

### 3.4. MAE

A total of 0.25 g of the initial grounded material was subjected to extraction in 20 mL of aqueous methanol in varying concentrations (40, 60, 70, and 80% *w*/*w*) at 40, 70, or 90 °C using an ETHOS X microwave oven (Milestone, Sorisole, Italy). The extraction process was performed at atmospheric pressure, following a heating–cooling cycle that involved heating for 1 min, followed by extraction for 10 min, and cooling for 10 min. Each sample underwent duplicate extraction under identical conditions. The resultant extracts were filtered through Whatman filter paper No. 1 using a Büchner funnel, and the filtrates were collected in a volumetric flask. The collected filtrates were then subjected to an evaporation process and subsequently treated as reported in Section 3.3.

### 3.5. ASE

Phenolic compounds of oregano samples were isolated using an ASE device (Dionex Corporation, ASE™ 350, Thermo Fisher Scientific Inc., Sunnyvale, CA, USA). A mixture of 1 g of herbal samples and approximately 6 mg of diatomaceous earth was filled in 22 mL cells, each equipped with a stainless-steel frit and a cellulose filter (diameter 27 mm, type D28, Thermo Fisher Scientific Inc., Sunnyvale, CA, USA). Extracts were prepared using aqueous methanol in varying concentrations (40, 60, 70, and 80% *w*/*w*) as a solvent and varying temperatures of 80, 120, or 140 °C under 1500 psi. The preheating time was 6 min, followed by the application of three extraction cycles with a total run time of 33 min, and 65% volume flush. The cells were purged with N_2_ gas for 90 s, and approximately 60 mL of each extract was collected. The collected extracts were subjected to filtration, followed by an evaporation process, and subsequently treated as reported in Section 3.3.

### 3.6. LC-MS Analysis

The major components of oregano extracts were quantified using a Shimadzu Nexera HPLC system with a diode array detector and a single quadrupole mass spectrometer (LCMS-2020) featuring an electrospray ionization (ESI) interface. Separations were performed on a Poroshell 120 EC-C_18_ analytical column (4.6 × 150 mm, 4 µm), following the method described by Irakli et al. [45]. Lab Solutions LC-MS Software was used for data acquisition and processing (Shimadzu, Kyoto, Japan). By contrasting the retention times, UV profiles, and mass spectra of unknown peaks with those of standards, the major phenolic compounds were identified. Targeted selective ion monitoring (SIM) scanning was used for quantitative analysis. The quantification of salvianolic acid isomers was based on standard curves generated by salvianolic acid B. The results were obtained using calibration curves from standard solutions and presented as mg per g of freeze-dried extract. Each analysis was carried out thrice.

### 3.7. Determination of Total Phenolic Content, Total Flavonoid Content, and Antioxidant Activity

The determination of total phenolic content (TPC), total flavonoid content (TFC), and antioxidant activities of the oregano extracts was performed spectroscopically as described by Skendi et al. [17] in a previous study. The TPC, TFC, and antioxidant activities (ABTS, DPPH, and FRAP) were evaluated as mg gallic acid (GAE), mg catechin (CATE), and mg Trolox equivalents (TE), respectively, per g of dried extract.

### 3.8. Statistical Analysis

All experimental values are reported as mean ± standard deviations of three independent measurements and subjected to two-way analysis of variance (ANOVA) to examine the effect of the extraction method (MAE, UAE, and ASE) and the oregano accessions (A1, A2, A3, A4, C1, and C2) according to the generalized linear model, using Tukey’s test at a significance level α = 0.05. All statistical analyses were conducted using Minitab software, version 18 (Minitab, Inc., State College, PA, USA). Principal component analysis (PCA) and heatmap analysis were performed using the web tool Clustvis [46] for the visualization of clustering on multivariate data.

## 4. Conclusions

There is a growing interest in natural antioxidants and sustainable extraction processes that can replace traditional methods due to their lower energy consumption, lower solvent usage, and shorter extraction times. The biological activity of the extracted phytochemicals should be of high importance and depend on the extraction method, though. The phytochemical profile of Greek oregano, a widely used medicinal herb, acquired with novel extraction techniques was evaluated in the present study. The results confirmed that the most efficient extraction technique for obtaining phytochemical-rich oregano extract was MAE, followed by UAE and ASE. In fact, MAE extracts had the highest TPC and TFC values, followed by UAE, whereas the respective values for ASE extracts were lower. The main phenolic components in the Greek oregano extracts were successfully identified and quantified using LC-DAD-MS. The most abundant compounds that were identified and quantified were rosmarinic acid, salvianolic acid B, carvacrol, and vicenin-2, whereas minor components were salvianolic acid isomers, eriodictyol, taxifolin, naringenin, caffeic acid, aromadendrin, apigenin, luteolin, crypto-chlorogenic acid, and apigenin-7-O-glucoside. Differences in phenolic content and individual phenolic profile were detected among the oregano accessions examined. Thanks to the application of multivariate analysis, the selection of a superior phenolic extract from oregano accessions cultivated in Greece was feasible, with a high amount of phenolic components and high antioxidant activity. Significant differences in the phenolic content and antioxidant activity of the investigated extracts may be attributed to the extraction technique employed. Novel technologies should be focused on and intensified in order to extend the usefulness of plants as natural sources of phytochemicals, particularly in pharmaceutical and food applications.

## Figures and Tables

**Table 1:** Effect of UAE, MAE, and ASE on TPC, TFC, and antioxidant activity as evaluated by ABTS, DPPH, and FRAP values among the oregano accessions.

	Accessions	TPC (mg GAE/g)		TFC (mg CATE/g)		ABTS (mg TE/g)		DPPH (mg TE/g)		FRAP (mg TE/g)	
UAE	C1	187.6 ± 11.0 ^c^		317.8 ± 9.4 ^a^		450.1 ± 44.8 ^bc^		281.8 ± 21.4 ^bc^		363.1 ± 8.6 ^b^	
	C2	185.4 ± 5.5 ^c^		292.4 ± 19.0 ^b^		467.0 ± 18.5 ^b^		288.3 ± 3.4 ^b^		376.3 ± 15.9 ^b^	
	A1	187.5 ± 1.0 ^c^		295.6 ± 3.9 ^b^		416.4 ± 9.8 ^c^		251.9 ± 6.5 ^c^		356.8 ± 3.9 ^b^	
	A2	178.0 ± 4.4 ^c^		259.1 ± 1.7 ^c^		429.3 ± 2.1 ^bc^		287.0 ± 13.9 ^b^		317.5 ± 7.1 ^c^	
	A3	198.4 ± 4.8 ^b^		323.1 ± 7.3 ^a^		518.6 ± 26.6 ^a^		329.3 ± 33.2 ^a^		422.3 ± 11.6 ^a^	
	A4	214.0 ± 3.6 ^a^		294.0 ± 9.1 ^b^		556.6 ± 12.3 ^a^		311.1 ± 2.4 ^ab^		431.3 ± 19.0 ^a^	
MAE	C1	195.0 ± 1.9 ^b^		331.3 ± 9.4 ^a^		500.1 ± 11.5 ^c^		334.2 ± 7.2 ^b^		475.3 ± 3.7 ^b^	
	C2	208.7 ± 4.0 ^b^		306.2 ± 5.8 ^b^		504.9 ± 14.2 ^c^		358.0 ± 0.7 ^a^		483.9 ± 1.0 ^ab^	
	A1	200.3 ± 1.8 ^b^		323.8 ± 0.4 ^a^		446.8 ± 15.5 ^d^		326.5 ± 9.5 ^bc^		427.7 ± 2.0 ^c^	
	A2	202.3 ± 8.5 ^b^		272.8 ± 6.7 ^c^		490.3 ± 13.0 ^c^		309.1 ± 11.4 ^c^		425.9 ± 13.0 ^c^	
	A3	203.1 ± 10.9 ^b^		328.1 ± 6.1 ^a^		538.6 ± 18.0 ^b^		364.2 ± 13.9 ^a^		482.8 ± 8.5 ^ab^	
	A4	232.1 ± 20.8 ^a^		321.6 ± 6.9 ^a^		593.6 ± 21.3 ^a^		321.8 ± 17.7 ^bc^		493.3 ± 7.6 ^a^	
ASE	C1	178.3 ± 0.5 ^a^		234.7 ± 4.7 ^ab^		392.1 ± 2.3 ^b^		213.6 ± 12.6 ^c^		335.1 ± 1.1 ^c^	
	C2	176.2 ± 0.2 ^a^		240.0 ± 6.5 ^ab^		432.0 ± 5.1 ^ab^		210.0 ± 10.0 ^c^		317.5 ± 8.5 ^d^	
	A1	157.2 ± 1.4 ^b^		220.2 ± 2.0 ^bc^		388.3 ± 10.4 ^b^		218.1 ± 7.4 ^bc^		318.5 ± 1.4 ^d^	
	A2	156.0 ± 4.1 ^b^		210.1 ± 2.7 ^c^		388.3 ± 7.6 ^b^		230.9 ± 11.8 ^abc^		309.4 ± 5.6 ^d^	
	A3	172.9 ± 4.4 ^a^		246.2 ± 3.3 ^a^		442.6 ± 9.1 ^a^		242.7 ± 0.8 ^ab^		371.5 ± 2.1 ^b^	
	A4	182.9 ± 11.3 ^a^		228.8 ± 16.6 ^abc^		449.1 ± 41.0 ^a^		254.0 ± 7.8 ^a^		392.3 ± 7.5 ^a^	
	Two-way ANOVA										
		TPC		TFC		ABTS		DPPH		FRAP	
Source	DF	F-value	*p*-value	F-value	*p*-value	F-value	*p*-value	F-value	*p*-value	F-value	*p*-value
Method (M)	2	104.93	<0.001	303.17	<0.001	113.27	<0.001	196.80	<0.001	954.29	<0.001
Accession (A)	5	20.15	<0.001	27.56	<0.001	44.21	<0.001	9.30	<0.001	131.31	<0.001
M × A	10	2.60	0.017	2.66	0.015	2.64	0.016	3.16	0.005	10.10	<0.001

Two-way ANOVA followed by Tukey’s multiple comparisons test explored differences between the oregano accessions and extraction methods; different letters in the same column for each extraction method (ASE, MAE, UAE) indicate significant differences (*p* < 0.05) amongst the means.

**Table 2:** Main values of major and minor phenolic compounds of oregano extracts obtained by three advanced extraction techniques.

				Two-Way ANOVA					
Phenolic Compounds	UAE	MAE	ASE	Method (M)		Accession (A)		M × A	
				DF = 2		DF = 5		DF = 10	
				F-Value	*p*- Value	F- Value	*p*- Value	F- Value	*p*- Value
Rosmarinic acid	165.21 ± 27.51 ^a^	184.95 ± 24.20 ^a^	132.71 ± 22.72 ^b^	278.26	<0.001	114.42	<0.001	9.39	<0.001
Salvianolic acid B	158.71 ± 17.51 ^a^	189.31 ± 16.89 ^b^	121.75 ± 20.53 ^c^	619.62	<0.001	62.28	<0.001	17.08	<0.001
Carvacrol	30.21 ± 10.35 ^a^	33.20 ± 10.23 ^a^	19.35 ± 9.75 ^b^	812.15	<0.001	373.27	<0.001	151.91	<0.001
Vicenin	32.93 ± 5.73 ^b^	43.83 ± 5.57 ^a^	31.49 ± 4.13 ^b^	255.48	<0.001	49.68	<0.001	14.48	<0.001
Salvianolic acid I	14.44 ± 7.86 ^b^	19.91 ± 4.83 ^a^	3.27 ± 1.28 ^c^	682.63	<0.001	250.34	<0.001	29.36	<0.001
Salvianolic acid II	4.12 ± 2.94 ^b^	10.91 ± 3.65 ^a^	6.50 ± 2.74 ^b^	349.33	<0.001	231.14	<0.001	26.69	<0.001
Salvianolic acid III	9.44 ± 2.25 ^b^	13.28 ± 1.48 ^a^	7.16 ± 1.52 ^c^	309.91	<0.001	20.25	<0.001	15.54	<0.001
Eriodictyol	4.69 ± 1.24 ^b^	7.28 ± 0.90 ^a^	4.90 ± 1.30 ^b^	563.32	<0.001	130.40	<0.001	35.05	<0.001
Taxifolin	2.00 ± 0.78 ^b^	3.09 ± 0.74 ^a^	1.40 ± 0.60 ^c^	574.52	<0.001	103.69	<0.001	57.61	<0.001
Naringenin	1.63 ± 0.77 ^b^	2.34 ± 0.60 ^a^	1.51 ± 0.67 ^b^	335.96	<0.001	402.12	<0.001	15.27	<0.001
Caffeic acid	0.61 ± 0.12 ^c^	1.05 ± 0.25 ^b^	1.78 ± 0.48 ^a^	1164.67	<0.001	127.61	<0.001	29.28	<0.001
Aromadendrin	0.60 ± 0.32 ^a^	0.95 ± 0.24 ^a^	0.50 ± 0.28 ^a^	145.43	<0.001	102.09	<0.001	2.59	0.018
Apigenin	0.32 ± 0.18 ^b^	0.50 ± 0.10 ^a^	0.38 ± 0.11 ^b^	90.23	<0.001	76.03	<0.001	6.63	<0.001
Luteolin	0.34 ± 0.09 ^b^	0.54 ± 0.09 ^a^	0.56 ± 0.11 ^a^	206.76	<0.001	23.02	<0.001	19.21	<0.001
c-chlorogenic acid	0.62 ± 0.43 ^ab^	0.76 ± 0.50 ^a^	0.37 ± 0.30 ^b^	545.42	<0.001	1398.41	<0.001	39.96	<0.001
Apigenin-7-O-glucoside	0.25 ± 0.17 ^a^	0.34 ± 0.19 ^a^	0.21 ± 0.15 ^a^	96.48	<0.001	327.91	<0.001	5.22	<0.001

Two-way ANOVA explored differences between the oregano accessions and extraction methods; different letters in the same row for each phenolic compound indicate significant differences (*p* < 0.05) amongst the extraction methods according to Tukey’s test; DF, degrees of freedom.

**Table 3:** Quantification of minor phenolic compounds in extracts of four oregano assessions (A1, A2, A3, A4) and two commercial cultivations (C1, C2) obtained by three advanced extraction techniques.

Accessions	Salvianolic Acid I			Salvianolic Acid II			Salvianolic Acid III		
	UAE	MAE	ASE	UAE	MAE	ASE	UAE	MAE	ASE
C1	13.10 ± 0.30	13.97 ± 0.04	2.92 ± 0.09	6.86 ± 0.43	9.58 ± 0.42	5.08 ± 0.08	12.96 ± 0.96	14.70 ± 0.70	7.69 ± 0.49
C2	19.40 ± 0.90	20.59 ± 1.19	3.45 ± 0.15	1.50 ± 0.15	12.06 ± 0.09	6.13 ± 0.17	7.60 ± 0.60	12.28 ± 1.05	5.60 ± 0.40
A1	2.19 ± 0.11	14.54 ± 0.64	0.87 ± 0.04	1.64 ± 0.18	9.75 ± 0.25	5.34 ± 0.16	8.94 ± 0.06	14.17 ± 1.30	5.58 ± 1.01
A2	24.17 ± 0.13	25.72 ± 0.72	3.89 ± 0.11	0.74 ± 0.11	4.83 ± 0.17	2.76 ± 0.16	11.25 ± 1.25	13.82 ± 0.82	6.47 ± 0.47
A3	7.81 ± 0.19	19.15 ± 1.15	3.61 ± 0.39	6.69 ± 0.31	13.50 ± 1.50	10.65 ± 0.35	6.87 ± 0.13	13.75 ± 0.25	9.35 ± 0.62
A4	20.00 ± 2.00	25.50 ± 0.50	4.88 ± 0.43	7.32 ± 0.23	15.78 ± 1.78	9.12 ± 1.12	9.00 ± 1.00	10.97 ± 0.03	8.27 ± 0.25
	**Eriodictyol**			**Taxifolin**			**Naringenin**		
	**UAE**	**MAE**	**ASE**	**UAE**	**MAE**	**ASE**	**UAE**	**MAE**	**ASE**
C1	4.54 ± 0.14	7.20 ± 0.20	4.16 ± 0.16	1.46 ± 0.06	2.73 ± 0.13	1.40 ± 0.10	1.70 ± 0.14	2.63 ± 0.13	1.51 ± 0.11
C2	4.10 ± 0.10	5.86 ± 0.03	3.36 ± 0.14	1.73 ± 0.08	2.20 ± 0.03	1.18 ± 0.08	1.61 ± 0.11	2.09 ± 0.02	1.05 ± 0.05
A1	4.74 ± 0.06	8.67 ± 0.47	6.58 ± 0.58	1.49 ± 0.17	2.52 ± 0.28	1.31 ± 0.19	1.41 ± 0.11	2.45 ± 0.09	1.28 ± 0.08
A2	4.46 ± 0.16	6.77 ± 0.29	3.69 ± 0.11	3.14 ± 0.14	4.32 ± 0.30	0.69 ± 0.06	1.28 ± 0.08	1.76 ± 0.09	1.11 ± 0.11
A3	3.17 ± 0.41	7.65 ± 0.15	5.41 ± 0.37	1.24 ± 0.22	3.58 ± 0.08	1.22 ± 0.11	0.65 ± 0.10	1.73 ± 0.13	1.18 ± 0.01
A4	7.12 ± 0.12	7.53 ± 0.13	6.20 ± 0.20	2.95 ± 0.10	3.17 ± 0.03	2.59 ± 0.17	3.11 ± 0.09	3.41 ± 0.21	2.93 ± 0.08
	**Aromadendrin**			**Apigenin**			**Luteolin**		
	**UAE**	**MAE**	**ASE**	**UAE**	**MAE**	**ASE**	**UAE**	**MAE**	**ASE**
C1	0.61 ± 0.05	0.99 ± 0.04	0.50 ± 0.02	0.38 ± 0.02	0.55 ± 0.01	0.43 ± 0.03	0.33 ± 0.03	0.67 ± 0.09	0.74 ± 0.04
C2	0.48 ± 0.19	0.82 ± 0.17	0.28 ± 0.02	0.20 ± 0.01	0.36 ± 0.01	0.26 ± 0.02	0.44 ± 0.02	0.49 ± 0.01	0.50 ± 0.02
A1	0.26 ± 0.05	0.75 ± 0.10	0.28 ± 0.04	0.26 ± 0.04	0.48 ± 0.02	0.42 ± 0.06	0.41 ± 0.05	0.52 ± 0.01	0.58 ± 0.07
A2	0.79 ± 0.05	1.19 ± 0.06	0.59 ± 0.04	0.28 ± 0.04	0.46 ± 0.02	0.28 ± 0.02	0.29 ± 0.03	0.50 ± 0.02	0.46 ± 0.04
A3	0.31 ± 0.01	0.72 ± 0.07	0.30 ± 0.03	0.15 ± 0.03	0.50 ± 0.02	0.34 ± 0.03	0.19 ± 0.01	0.63 ± 0.03	0.64 ± 0.01
A4	1.15 ± 0.12	1.30 ± 0.10	1.06 ± 0.11	0.64 ± 0.04	0.67 ± 0.11	0.55 ± 0.09	0.37 ± 0.02	0.45 ± 0.05	0.47 ± 0.04
	**Caffeic acid**			**Crypto-chlorogenic acid**			**Apigenin-7-O-glucosie**		
	**UAE**	**MAE**	**ASE**	**UAE**	**MAE**	**ASE**	**UAE**	**MAE**	**ASE**
C1	0.43 ± 0.02	0.71 ± 0.01	1.42 ± 0.22	1.46 ± 0.04	1.72 ± 0.03	0.98 ± 0.02	0.49 ± 0.02	0.57 ± 0.02	0.37 ± 0.02
C2	0.63 ± 0.03	0.95 ± 0.05	1.69 ± 0.07	0.58 ± 0.06	0.64 ± 0.09	0.23 ± 0.01	0.19 ± 0.01	0.26 ± 0.01	0.17 ± 0.01
A1	0.72 ± 0.02	1.40 ± 0.03	2.53 ± 0.06	0.60 ± 0.02	0.85 ± 0.05	0.50 ± 0.05	0.47 ± 0.06	0.61 ± 0.08	0.44 ± 0.04
A2	0.59 ± 0.04	0.89 ± 0.02	1.09 ± 0.09	0.27 ± 0.01	0.36 ± 0.02	0.19 ± 0.01	0.15 ± 0.01	0.29 ± 0.03	0.09 ± 0.01
A3	0.51 ± 0.09	1.10 ± 0.12	1.93 ± 0.01	0.15 ± 0.01	0.18 ± 0.02	0.09 ± 0.01	0.11 ± 0.01	0.18 ± 0.02	0.11 ± 0.02
A4	0.77 ± 0.03	1.29 ± 0.08	2.04 ± 0.06	0.65 ± 0.03	0.78 ± 0.02	0.26 ± 0.01	0.10 ± 0.01	0.13 ± 0.02	0.09 ± 0.02

## Data Availability

Data are contained within the article.

## Supplementary Materials

The following supporting information can be downloaded at: https://www.mdpi.com/article/10.3390/plants13213087/s1, Table S1. Pearson’s correlation coefficients among antioxidants and phenolic components of oregano extracts.
