# Supplementary material for: Characterization of Natural Bioactive Compounds from Greek Oregano Accessions Subjected to Advanced Extraction Techniques
Source: Plants (Basel). 2024 Nov 2;13(21):3087. doi: 10.3390/plants13213087 (PMC11548386; doi:10.3390/plants13213087)
Supplement: Supplementary file 1 [file plants-13-03087-s001.zip › plants-3272439-supplementary.pdf]

Table S1. Pearson's correlation coefficients among antioxidants and phenolic components of oregano extracts.

	**TPC**	**TFC**	**RMA**	**SALB**	**CAR**	**VIC**	**SALI**	**SALII**	**SALIII**	**ERY**	**TAX**	**NAR**	**ARO**	**API**	**LUT**	**CA**	**cCLA**	**APGL**	**ABTS**	**DPPH**	**FRAP**
TPC	1.00																				
TFC	**0.734**	1.000																			
RMA	**0.559**	**0.807**	1.000																		
SALB	**0.754**	**0.854**	**0.849**	1.000																	
CAR	**0.591**	**0.362**	0.289*	0.268*	1.000																
VIC	**0.665**	**0.334**	0.280*	**0.570**	0.231^{ns}	1.000															
SALI	0.302*	**0.478**	0.270*	0421**	0.421**	**0.445**	1.000														
SALII	**0.675**	**0.461**	**0.453**	**0.424**	**0.731**	0.145^{ns}	0.416**	1.000													
SALIII	0.512**	**0.646**	**0.546**	**0.770**	**0.445**	**0.800**	**0.543**	0.379**	1.000												
ERY	**0.505**	0.404**	0.403***	**0.601**	0.169^{ns}	**0.679**	**0.491**	**0.649**	**0.619**	1.000											
TAX	**0.629**	0.414**	0.281*	**0.493**	**0.466**	**0.713**	**0.824**	0.368**	**0.687**	**0.681**	1.000										
NAR	**0.640**	0.388**	0.378**	0.372*	0.383**	0.344*	**0.660**	**0.654**	**0.421**	**0.718**	**0.566**	1.000									
ARO	**0.653**	0.313*	**0.215**	0.322*	**0.530**	**0.526**	**0.838**	**0.495**	**0.548**	**0.622**	**0.791**	**0.819**	1.000								
API	**0.534**	0.246^{ns}	0.275*	0.284*	0.242^{ns}	**0.477**	**0.524**	**0.713**	**0.422**	**0.809**	**0.601**	**0.860**	**0.765**	1.000							
LUT	**-0.145**	-0.210^{ns}	0.134^{ns}	0.046^{ns}	-0.112^{ns}	0.328*	-0.256^{ns}	0.244^{ns}	0.131^{ns}	0.356**	0.033^{ns}	0.120^{ns}	-0.007^{ns}	0.374**	1.000						
CA	**-0.374**	**-0.587**	-0.368**	-0.365*	**-0.469**	-0.141^{ns}	-0.400*	**0.180**	-0.374**	0.218^{ns}	-0.200^{ns}	0.027^{ns}	-0.177^{ns}	0.228^{ns}	**0.517**	1.000					
cCLA	0.227^{ns}	**0.440**	**0.587**	**0.465**	0.135^{ns}	0.396**	0.093^{ns}	**0.138**	**0.460**	0.255^{ns}	0.057^{ns}	0.421**	0.247^{ns}	**0.723**	0.212^{ns}	-0.304*	1.000				
APGL	-0.057^{ns}	0.303*	**0.437**	**0.487**	-0.249^{ns}	**0.521**	-0.235^{ns}	-0.144^{ns}	0.417**	0.327*	-0.021^{ns}	0.071^{ns}	-0.098^{ns}	0.297*	-0.163^{ns}	-0.059^{ns}	**0.723**	1.000			
ABTS	**0.885**	**0.722**	**0.517**	**0.534**	**0.647**	0.169^{ns}	**0.721**	**0.718**	0.401**	**0.450**	**0.575**	**0.560**	**0.594**	**0.493**	-0.173^{ns}	**-0.525**	0.094^{ns}	-0.245^{ns}	1.000		
DPPH	**0.761**	**0.841**	**0.656**	**0.797**	**0.504**	**0.448**	**0.665**	**0.520**	**0.684**	**0.485**	**0.600**	0.389*	**0.496**	0.397*	-0.163^{ns}	**-0.523**	0.189^{ns}	0.062^{ns}	**0.784**	1.000	
FRAP	**0.830**	**0.764**	**0.731**	**0.774**	**0.514**	0.383**	**0.610**	**0.759**	**0.628**	**0.647**	**0.607**	**0.619**	**0.601**	**0.531**	0.086^{ns}	**-0.252**	0.259^{ns}	0.049^{ns}	**0.833**	**0.844**	1.000

* and **, significant difference at $p \leq 0.05$ and $p \leq 0.01$, respectively; values in bold, significant difference at $p \leq 0.001$; ^{ns}, non-significant at $p \geq 0.05$
